# The healthcare seeking behaviour of adult patients with asthma at Chitungwiza Central Hospital, Zimbabwe

**DOI:** 10.1186/s40733-020-00060-y

**Published:** 2020-08-12

**Authors:** Pisirai Ndarukwa, Moses J. Chimbari, Elopy N. Sibanda, Tafadzwa Madanhire

**Affiliations:** 1 University of KwaZulu-Natal, College of Health Sciences, School of Nursing and Public Health, Durban, South Africa; 2 Asthma, Allergy and Immune Dsyfunction Clinic, 113 Kwame Nkrumah Ave, Harare, Zimbabwe; 3 Biomedical Research and Training Institute, 11 Routledge Avenue, Milton Park, Harare, Zimbabwe

**Keywords:** Asthma, Health seeking behaviour, Zimbabwe

## Abstract

**Background:**

Although asthma is a serious public health concern in Zimbabwe, there is lack of information regarding the decision to seek for healthcare services among patients. This study aimed to determine the health care seeking behaviour of adult patients with asthma attending Chitungwiza Central Hospital in Zimbabwe.

**Methods:**

A cross-sectional study was conducted among 400 patients with asthma. A questionnaire with four thematic areas (i) patients’ demographic characteristics, (ii) types of health seeking behaviours (iii) knowledge of asthma treatment and (iv) attitudes on asthma treatment was used.

**Results:**

We determined the sequence of remedial action that people undertake to rectify perceived ill health commonly referred to as health care seeking behaviours in 400 adult patients with asthma. This behaviour was considered good if the patient sought care at the hospital/clinic and or private practitioners. Poor health seeking behaviour was adjudged if patients sought no treatment, self-treated or resorted to traditional or faith healers for care.

The majority 261(65.3%) of the study participants were females mainly between ages 29–39 years who lived in the urban setting. Distance to health facility, perception of supportive roles of healthcare providers, perceived good quality of service and knowledge of asthma complications were key determinants for health seeking behaviour. The results showed that majority 290 (72.5%) reported good health seeking behaviour. The correlates of good health seeking behaviour included financial capacity to pay for medical care [OR: 0.50 (CI: 0.31–0.83); *p* = 0.008)] and receiving good quality of asthma treatment [OR: 0.59 (CI: 0.37–0.93); *p* = 0.03)]. The inability to voluntarily seek own asthma treatment [OR: 1.68 (CI: 1.05–2.70); *p* = 0.03) was a significant risk factor (68% more likely) for poor health seeking behaviour.

**Conclusions:**

We concluded that prior to scaling up asthma treatment programmes in Zimbabwe, there is need to address, individual-level, community-level and health service level barriers to health seeking among asthma patients.

## Background

Asthma is a serious public health problem worldwide particularly in low and middle income countries [1]. Asthma affects 358 million people globally and more than 300,000 deaths are registered yearly. More negative impacts of asthma are experienced in poorly resourced countries especially those in the Sub Saharan Africa (SSA) [2]. In SSA and South Asia, severe asthma is reported to be common because of poor access to medication and poor health care seeking behaviour that compromises the timely provision of health care services [1, 3].

Health care seeking behaviour is defined as a sequence of remedial action that people undertake to rectify perceived ill health [4]. The treatment of asthma is reportedly closely linked to health care seeking behaviour [5]. The time span from symptoms onset to contacting a health care provider, the type of health care provider chosen and the patient’s compliance with treatment reflects on their health care seeking behaviour [4]. The United Kingdom National Institute for Clinical Excellence (NICE) reported that patients’ perceptions (e.g., beliefs about and experiences of the illness and treatment) and practicalities (e.g., resources and access to treatment) are important determinants of health care seeking behaviours [6]. A study in Nepal has shown that ethnicity, religion and type of health problems were significantly associated with the health care seeking behaviour [3].

A study by Kuuire et al. in Ghana showed that patients who are poor were less likely to seek health care [5]. Several other studies [7–10] on health care seeking behaviours have been conducted in Zimbabwe, but none focused on asthma. These studies have reported that religion, distance to health care facility, health care worker attitudes towards patients and work, the availability and affordability of medication, patients’ knowledge about their conditions were associated with delays in seeking health care services [7–10].

Good health care seeking behaviour practices among asthma could potentially reduce the morbidity and mortality rates. Additionally, public health interventions need to be informed by the health care seeking behaviour among asthma patients. However, there are no studies which have determined health care seeking behaviours related to asthma in Chitungwiza to the authors’ knowledge.

The objective of this study was to determine the health care seeking behaviour by adult patients with asthma at Chitungwiza Central Hospital.

## Methods

### Study design and setting

We conducted a cross-sectional study to determine the health care seeking behavior related to adult patients with asthma at Chitungwiza Central Hospital using a questionnaire. The study site was Chitungwiza Central Hospital which is situated about 30 km south east of Harare, Zimbabwe. The hospital has a bed capacity of 500 beds including general, specialised, maternity and emergency care beds. It also has a casualty department which manages patients who present with acute asthmatic attacks. The high dependence unit manages more complex cases of asthma such as status asthmatics.

### Data collection

The Kobo Collect Toolbox was used for data collection. The KoBo Collect, is an open source platform that is used to collect and analyze data [11]. The research assistants included six (6) nurses who were working in the outpatient department and had been assigned to the study by the hospital management based on their willingness. Data collectors were trained on how to conduct interviews using the Kobo Collect Toolbox before the actual data collection was initiated. Data were collected from 28 November 2018 to 15 December 2018. All study participants were interviewed in an outpatient clinic.

Participants were interviewed (face-to face) at the outpatient clinic, casualty department or in the medical wards. All asthmatic patients who were admitted to the medical wards due to their asthma, those coming for nebulization at the emergency department following asthmatic attack and those coming for their routine asthma care services in the outpatient department (Pulmonary care clinic) and who agreed to participate in this study were interviewed. Health care seeking behavior questions in the questionnaire (see supplementary file 1) were developed according to the Mediar Health seeking survey [12] while the rest of the questions were based on the World Health Organisation (WHO) Stepwise tool [13]. The questionnaire was adapted and modified to align with objectives of the study.

We used the Medical Research Council of Zimbabwe (MRCZ) approved translator translated questionnaire from English to the local Shona language. The questionnaire was pretested with 30 inpatients (not part of the main study) who were admitted at South Medical Hospital in Chitungwiza. Based on the pretest of the questionnaire, we modified the questions that were perceived to be vague or too sensitive. The face to face interviews were done mainly using the local Shona language version of the questionnaire. Although during the pre-test it was evident that participants preferred the Shona version, the English version of the questionnaire was also available and offered to the participants who preferred that. The questionnaire was divided into four sections: (i) demographic information (ii) health care seeking behaviors (iii) knowledge on asthma and (iv) attitudes about asthma. In this study health seeking behaviour was defined as the action undertaken by the study participants to seek appropriate treatment for asthma.

### Sample size determination and sampling techniques

We calculated a sample size of 400. When n is the sample size, z is the standard normal variable, p is the expected proportion in the population and e is the absolute error or precision. The formula below was used to determine the sample size

$$ n=\frac{z^2 pq}{e^2}. $$

Assuming an error margin of 5% at 95% confidence interval a sample size of 384 was calculated using the Dobson formula: $n=\frac{z^2 pq}{e^2}$ and this was adjusted to 400 to cater for attrition. The sample included patients who reported to outpatients for routine check-up for asthma. We therefore interviewed these patients with asthma to determine their healthcare seeking behaviours at Chitungwiza Central Hospital.

### Statistical analysis

We used Stata (StataCorp. 2017. Stata Statistical Software: Release 15. College station, TX: StataCorp) to perform the analyses. Descriptive statistics on demographic characteristics were analyzed as frequencies and proportions. Statistical significance was determined at *p*-value < 0.05. Bivariate and multiple logistic regression was used to identify significant risk factors (*p* < 0.05) for health seeking behaviour. The responses to attitudes on asthma treatments were presented on a bar graph for different questions on attitudes.

### Ethical considerations

Permission to conduct the study was given by the Biomedical Research Ethics Committee of the University of KwaZulu-Natal (BE613/18) and also by Medical Research Council of Zimbabwe (A/2352). Gatekeepers’ permission was granted by Ministry of Health and Child Care and Chitungwiza Central Hospital. The community advisory board for Chitungwiza allowed the study to be conducted. We also got the written informed consent from the study participants. Data captured through mobile electronic devices did not capture personal identifying information for the patient and only computer generated codes were used for each participant record.

## Results

### Socio-demographic characteristics

Table 1 below summarized the characteristics of the 400 asthma participants at Chitungwiza Central Hospital. Two hundred and sixty-one (65.3%) were females. The ages of participants ranged from 18 years to 91 years. The majority [142, (35.5%)] of the participants were aged 29 years to 39 years whilst those aged 18–28 years were 125(31.3%). The majority [159, (39.8%)] of the participants attained a secondary level of education. Those who had attained a college level of education were 127 (31.8%), of whilst those who attained a university level of education were 54 (13.5%), 60 (15%) of the participants had attained primary school level of education. Just above half [201, (50.2%)] of asthma patients were employed while 199 (49.8%) were unemployed. Many 247 (61.8%) were married while those divorced were 18(4.5%). Most [163, (40.8%)] were of a Pentecostal religion while 100 (25%) belonged to apostolic religion. Majority [276, (69%)] stayed in the urban setting and 58 (14.5%) were from the rural setting.

**Table 1:** Demographic characteristics *N* = 400

Variable	Frequency (N)	Percentage (%)
Gender		
Males	139	34.7
Females	261	65.3
Age groups		
18–28	125	31.3
29–39	142	35.5
40–50	70	17.5
51–60	29	7.2
61+	34	8.6
Level of Education		
Primary	60	15
Secondary	159	39.8
College	127	31.8
University	54	13.5
Employment status		
Employed	201	50.2
Unemployed	199	49.8
Marital Status		
Married	247	61.8
Cohabiting	4	1.0
Single	94	23.5
Widowed	37	9.3
Divorced	18	4.5
Religion		
Apostolic	100	25
Pentecostal	163	40.8
Protestants	79	19.8
Others	58	14.5
Area of residence		
Urban	276	69
Peri-urban	42	10.5
Rural	58	14.5
Farms	24	6

### Risk factors associated with health care seeking behaviour of adult patients with asthma

The time taken to reach the health facility or the preferred place of treatment was categorized to identify asthma patients who took less than 30 min, between 30 min and 1 h and more than 1 h. Nearly half 195 (48.8%) indicated to have spent between 30 min and 1 h to reach their preferred place of treatment. Majority 290(72.5%) reported good health seeking behaviours (private prictitioners and hospital/clinc) whilst 110(27.5%) reported poor health seeking behaviour (self-treatment, traditional healer, none treatment and faith healers). After multiple logistic regression, participants who reported that they able to pay for asthma medication were 50% less likely to have poor health seeking behaviour. Those participants who reported that they did not receive good quality of asthma treatment were 41% less likely to have poor health seeking behaviour. The participants who reported inability to voluntarily seek own asthma treatment were 68% more likely to have poor health seeking behaviour (See Table 2).

**Table 2:** Risk factors associated with health seeking behaviour of asthma patients

Variable	Total	Health seeking behaviour		cOR (95% CI)	***P***-value	aOR (95%CI)	***P***-value
		Good (%)	Poor (%)				
**Time taken to reach place of treatment**							
Less than 30 min	112 (28)	80 (71.4)	32 (28.6)	1^r^			
30 min-1 h	195 (48.8)	149 (76.4)	46 (23.6)	0.77 (0.46–1.31)	0.34		
More than 1 h	93 (23.2)	61 (65.6)	32 (34.4)	1.31 (0.73–2.37)	0.37	–	–
**Distance**							
Less than 5 km	114 (28.5)	84 (73.7)	30 (26.3)	1^r^			
5-10 km	140 (35)	112 (80)	28 (20)	0.7 (0.39–1.26)	0.23		
11-15 km	68 (17)	44 (64.7)	24 (35.3)	1.53 (0.80–2.92)	0.20	–	–
More than 15 km	78 (19.5)	50 (64.1)	28 (35.9)	1.57 (0.84–2.92)	0.16		
Financial capacity to pay for medication							
No	217 (54.3)	152 (70.1)	65 (29.9)	1^r^		1^r^	
Yes	183 (45.7)	138 (75.4)	45 (24.6)	0.76 (0.49–1.19)	0.23	0.50 (0.31–0.83)	0.008*
Good relation with health care provider							
No	14 (3.5)	10 (71.4)	4 (28.6)	1^r^			
Yes	386 (96.5)	280 (72.5)	106 (27.5)	0.95 (0.29–3.08)	0.93	–	–
Health care providers supportive of asthma treatment							
No	196 (49)	152 (77.6)	44 (22.4)	1^r^			
Yes	204 (51)	138 (67.7)	66 (32.3)	1.65 (1.05–2.58)	0.03*	–	–
Good quality of asthma treatment							
Yes	229 (57.3)	156 (68.1)	73 (31.9)	1^r^		1^r^	
No	171 (42.7)	134 (78.4)	37 (21.6)	0.59 (0.37–0.93)	0.02*	0.59 (0.37–0.95)	0.03*
Seek permission for asthma treatment							
Yes	103 (25.7)	78 (75.7)	25 (24.3)	1^r^			
No	297 (74.3)	212 (71.4)	85 (28.6)	1.25 (0.75–2.10)	0.40	–	–
Voluntarily seek own asthma treatment							
Yes	222 (55.5)	175 (78.8)	47 (21.2)	1^r^		1^r^	
No	178 (44.5)	115 (64.6)	63 (35.4)	2.04 (1.31–3.18)	0.002*	1.68 (1.05–2.70)	0.03
Knowledge of asthma symptoms							
Knowledgeable	216 (54)	148 (68.5)	68 (31.5)	1^r^			
Partial knowledge	180 (45)	139 (77.2)	41 (22.8)	0.64 (041–1.01)	0.05	–	–
Not knowledgeable	4 (1)	3 (75)	1 (25)	0.73 (0.07–7.10)	0.78	–	–
Knowledge of asthma complications							
Yes	230 (57.5)	153 (66.5)	77 (33.5)	1^r^			
No	170 (42.5)	137 (80.6)	33 (19.4)	0.48 (0.30–0.76)	0.002*	–	–

*1*^*r*^: reference category, p-value: * significant p-value, − insignificant (p > 0.05) in multiple logistic regression

### Asthma knowledge and perceptions

Majority (85.6%) of the study participants indicated that asthma symptoms were reversible. The participants’ attitude on whether or not asthma is a rare illness were divergent. Fifty two point 8% (52.8%) of the participants disagreed with the statement that asthma treatment is not safe for patients. Almost all the participants (93.3%) agreed that diagnosing and treating asthma was costly. Slightly more than half of the participants (51.5%) said they were not aware that asthma could be treated using traditional medicines.

Attitudes:

(define these and report what was observed, the table below has not been introduced or summarised)

**Fig. 1:**
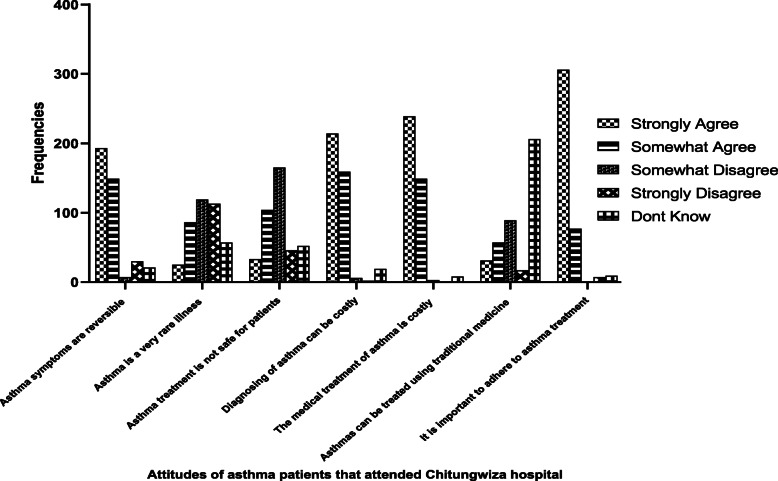
Attitudes of asthma patients that attended

Almost all the participants (95.8%) agreed on the importance of adhering to asthma treatments (Fig. 1).

## Discussion

To the authors’ knowledge, health seeking behaviour of patients with asthma has been scarcely investigated in Zimbabwe. This cross sectional study found that health seeking behavior of asthma patients was influenced by the interplay between factors that included perceived supportive roles of healthcare providers, perceived quality of treatment services offered and distance from the health care provider. Such results are consistent with previous literature from Uganda and Nigeria [14, 15]. Furthermore, our results concurred with Andersen’s [16] Behavioural Model of Health Utilisation whish stated that the service utilization is determine by the predisposing, enabling and need factors. Despite these findings, there were substantial factors which we found to hinder health seeking behaviour among asthma patients such as distance to the health facility.

Our study also revealed that most asthma patients who participated in this study were staying in an urban setting despite that Chitungwiza Central Hospital serves both urban and rural clientele. Such findings are important for a number of reasons in a resource constrained setting such as ours. First the trend could be as a result of the increasing socioeconomic difficulties affecting more of the rural population which could have resulted in less numbers affording health services. This therefore implores the need to strengthen public health service provision within these rural areas. Secondly, the trend could reflect the increasing rural to urban migration in our setting [17]. The World Health Organisation (WHO) has cited access to health emergency management as a factor influencing urbanization [18]. WHO [18] further noted that urbanization is a leading global trend which has a significant impact on public health which include a rise in non-communicable diseases like asthma. Thirdly, this finding might be tied to access of health care services as alluded to in the foregoing discussion regarding distance to the health facility. This finding concurred with Kuuire, et al. [5] who demonstrated that distance to health facility influenced health seeking behaviour.

Our study has shown that inability to voluntarily seek own asthma treatment is a significant contributor to poor health care seeking behaviour. Such a finding is very important in settings like ours where some individuals may need to seek the permission of spouses, religious leaders for the treatment of their illness. This therefore entreats the need to improve health service provision at community level. This has been corroborated by Dagnew et al. [19] who has suggested the need to improve health care services at community level to improve health care seeking behaviours.

Evidence from this study seems to suggest that asthmatic patients in Chitungwiza engage in medical pluralism as our results showed that some patients consulted religious healers as well as traditional healers. In light of such findings, it is important for further studies to unpack mechanisms and reasons for such health seeking behaviours. Such findings are however not peculiar to Chitungwiza as studies in other settings have also demonstrated that patients with chronic conditions tend to consult faith healers [20]. Multiple consultations of both the biomedical and traditional health practitioners may present serious challenges for patients with asthma which may include non-adherence and potentially adverse drug to drug interaction [19, 21]. There is therefore need to strengthen patient education programmes and raise community awareness of the dangers related to such practices.

Study respondents acknowledged the importance of adherence to asthma medication in achieving asthma control. A study by Eakin [22] indicated that patient education is the foundation for promoting adherence and effective self-management. However, our study did not establish medication adherence levels and future studies could potentially explore the interplay between adherence levels and the decision to seek treatment as monitoring patients’ adherence and providing feedback and reinforcement is a powerful behavioural strategy that can improve asthma self-management [22].

Evidence from our study seems to suggest that those patient with asthma who are able to pay for their medications were more likely to have good health seeking behaviour. Our findings concurred with findings from studies carried out in Ghana and Malaysia [5, 23] which have shown that income level is a significant predictor of health seeking behaviour.

## Conclusion

In conclusion, this study provides an understanding of health seeking behaviours of adult patients with asthma in a resource constrained setting and lay a crucial baseline for conducting larger studies whose results can be generalized across different settings within Zimbabwe. Such studies could also adopt qualitative methodologies aimed at explicating the motivations behind both individual level, community level and health system level factors affecting health seeking behaviour among adult patients with asthma.

## Supplementary information

**Additional file 1.**

## Data Availability

The data set analysed during the current study is available from the corresponding author on reasonable request.

## Abbreviations

BREC: Biomedical Research Ethics Committee
MRCZ: Medical Research Council of Zimbabwe
NICE: National Institute for Clinical Excellence
SSA: Sub-Saharan Africa
WHO: World Health Organisation
