# Supplementary material for: The healthcare seeking behaviour of adult patients with asthma at Chitungwiza Central Hospital, Zimbabwe
Source: Asthma Res Pract. 2020 Aug 12;6:7. doi: 10.1186/s40733-020-00060-y (PMC7424971; doi:10.1186/s40733-020-00060-y)
Supplement: Supplementary file 1 — Additional file 1. [file 40733_2020_60_MOESM1_ESM.docx]

**001 QUESTIONNAIRE IDENTIFICATION NUMBER |___|___|___|**

*Introduction:* ‘My name is _____________________ I am part of the research team and we are here to interview people here in [Name of City, region or site] in order to determine participants’ health care seeking behaviours on asthma treatment**.**

Confidentiality and consent: “I’m going to ask you some personal questions that some people find difficult to answer. Your answers are completely confidential. Your name will not be written on this form, and will never be used in connection with any information you tell me. You do not have to answer any questions that you do not want to answer, and you may end this interview at any time you want to. However, your honest answers to these questions will help us better understand what people think, say and in relation to **treatment of asthma**. We would greatly appreciate your help in responding to this survey. It will take about 30 minutes to ask the questions. Would you be willing to participate?’

Yes

No

*(Signature of interviewer certifying that informed consent have been given verbally by respondent)* …………………………………………………………………………………………………

***INTERVIEWER INFORMATION***

*Results codes:* completed (1) refused (2); partially completed (3); other (4)

002 INTERVIEWER: Code [____I____] Name_____________________________

003 DATE INTERVIEW: _____\_____\2018

CHECKED BY SUPERVISOR: Signature__________________ Date__________________

***SECTION A: Socio-demographic characteristics***

| **No** | **Questions and filters** | **Coding categories** | **Code** |
| --- | --- | --- | --- |
| A1 | How old are you at your last birthday | Age in completed years [____\|____] |  |
| A2 | Identify your gender | 1. MALE 2. FEMALE |  |
| A3 | What is your ethnic group? | 1. Shona 2. Tonga 3. Ndebele 4. Others (please specify) |  |
| A4 | Have you ever attended school? | 1. YES 2. NO |  |
| A5 | What is the highest level of school you completed:  CIRCLE ONE | 1. PRIMARY 2. SECONDARY 3. COLLEGE LEVEL 4. ABOVE COLLEGE LEVEL |  |
| A6 | Occupation of participant | 1. STUDENT 2. SELF EMPLOYED 3. EMPLOYED BY GOVERNMENT 4. PRIVATE COMPANY 5. HOUSEWIFE 6. RETIRED 7. NOT EMPLOYED | Describe further? |
| A7 | How many other people are employed in your family? | ………………………………………………….. |  |
| A8 | What type of job do they do? | ………………………………………………….. |  |
| A9 | What is the major source of family income? | ………………………………………………….. |  |
| A10 | How long have you lived in this area? | NUMBER OF YEARS \|__\|__\|  NUMBER OF MONTHS \|__\|__\| |  |
| A11 | Marital status | 1. MARRIED 2. DIVORCED 3. SINGLE (NEVER MARRIED) 4. WIDOWED 5. OTHER……………………………… |  |
| A12 | Religion | 1. Apostolic sect 2. Pentecostal 3. Protestants 4. Others (Please specifiy) |  |
| A13 | Area of residence | 1. Urban 2. Per urban 3. Rural 4. Farms |  |

***SECTION B: HEALTH CARE SEEKING BEHAVIOURS FOR ASTHMA TREATMENTS***

|  | **Questions** | **Categories** | **Codes** |
| --- | --- | --- | --- |
| B1 | Preferred source of treatment for asthma | 1.Self treatment  2.Privte medical practitioners  3. Traditional practitioners  4. Hospitals  5. No treatment  6.Other (specify)................................................... |  |
| B2 | Reason for not seeking treatment for asthma at the medical facility? | 1. Less severity of symptoms 2. Lack of money 3. Distance of health facility 4. Less belief in modern medicine 5. Lack of help/support 6. Pressure of work 7. Others |  |
| B3 | How long does it take you to reach a place you can get treatment for asthma? | 1. Less than 30 minutes 2. Between 30 minutes- 1hour 3. More than 1 hour |  |
| B4 | How long does it take you to reach a traditional healer/herbalist/spiritual healer for your asthma? | 1. Less than 30 minutes 2. Between 30 minutes- 1hour 3. More than 1 hour 4. I do not go to these healers. |  |
| B5 | Distance from health care faciltiy? | 1. Within 5km 2. 5-10kms 3. 10-15kms 4. Above 15km |  |
| B6 | Do you have adequate money to pay for your monthly supplies for asthma? | 1. Yes  2. No |  |
| B7 | Is your relationship with health care workers good? | 1. Yes  2. No |  |
| B8 | How do you rate the care you receive at this facility for asthma? | 1. Good 2. Average 3. Bad |  |
| B9 | Are the health care workers supportive for your treatment for asthma? | 1. Yes 2. No |  |
| B10 | If NO, how do you expect them to improve? | 1. Always available during acute asthmatic attack in the treatment room 2. They must inform us of what could cause the asthmatic attack for us to prevent it. 3. They must treat us with respect always during treatment. |  |
| B11 | What is quality of health care supply in the hospital good for asthma treatment | 1. Yes 2. No |  |
| B12 | Do you take your asthma medicines as prescribed for you? | 1. Yes 2. No |  |
| B13 | If NO, what could be the reason for failing to adhere? | 1. Medication are not available 2. Place where medicines are available is too far 3. Fear of side effects 4. This condition was as a result of having been bewitched 5. There was no advice to take medicines to treat asthma 6. Others…………………………………………………………………………………….. |  |
| B14 | Do you have a treatment supporter? | 1. Yes…………................................................  2. No…………….............................................. |  |
| B15 | If yes, who normally support you during treatment? | ...........................................................................  ........................................................................... |  |
| B16 | Do you keep pets at home? | 1. Yes 2. No |  |
| B17 | If YES, do you think that they can cause an exacerbation of your asthma attack? | 1. Yes 2. No |  |
| B18 | Do you think the services provided at the hospital are adequate for asthma treatment? | 1. Yes 2. No |  |
| B19 | Do you need to seek permission from someone before your seek treatment for asthma? | 1. Yes 2. No |  |
| B20 | Do you ordinarily go out of your own volition to seek for asthma treatment? | 1. Yes 2. No |  |

| **SECTION C. ASTHMA ATTITUDES**  **Now I’d like to ask you a series of questions to get a sense of how you might feel about Asthma treatment. For each statement I read, please tell me whether you strongly agree, somewhat agree, somewhat disagree or strongly disagree**. *DO NOT MENTION ‘DON’T KNOW’ OPTION, BUT IF PARTICIPANT SAYS ‘DON’T KNOW’, MARK THAT OPTION* | | | | | | |
| --- | --- | --- | --- | --- | --- | --- |
|  | | Strongly  Agree | Somewhat Agree | Somewhat Disagree | Strongly Disagree | Don’t Know |
| **C1** | **Asthma symptoms are reversible** | ❑ 1 | ❑ 2 | ❑ 3 | ❑ 4 | ❑ 88 |
| **C2** | **Asthma is a very rare illness** | ❑ 1 | ❑ 2 | ❑ 3 | ❑ 4 | ❑ 88 |
| **C3** | **Asthma treatment is not safe for patients** | ❑ 1 | ❑ 2 | ❑ 3 | ❑ 4 | ❑ 88 |
| **C4** | **Diagnosing of asthma can be costly** | ❑ 1 | ❑ 2 | ❑ 3 | ❑ 4 | ❑ 88 |
| **C5** | **The medical treatment of asthma is costly** | ❑ 1 | ❑ 2 | ❑ 3 | ❑ 4 | ❑ 88 |
| **C6** | **Asthmas can be treated using traditional medicine** | ❑ 1 | ❑ 2 | ❑ 3 | ❑ 4 | ❑ 88 |
| **C7** | **It is important to adhere to asthma treatment** | ❑ 1 | ❑ 2 | ❑ 3 | ❑ 4 | ❑ 88 |

**Any other information:**

________________________________________________________________________________________________________________________________________________________________________________________________________________________________________________________________________________________________

We have now come to the end of the questionnaire. Thank you for your time and patience. Your co-operation has been much appreciated.
